# Supplementary material for: Two-dimensional Thouless pumping of light in photonic moiré lattices
Source: Nat Commun. 2022 Nov 8;13:6738. doi: 10.1038/s41467-022-34394-3 (PMC9643509; doi:10.1038/s41467-022-34394-3)
Supplement: Supplementary file 1 — Supplementary Information [file 41467_2022_34394_MOESM1_ESM.pdf]

Two-dimensional Thouless pumping of light in photonic moiré lattices

Peng Wang,[1] Qidong Fu,[1] Ruihan Peng,[1] Yaroslav V. Kartashov,[2,3] Lluis Torner,[2,4] Vladimir V. Konotop,[5] Fangwei Ye[1*]

[1] *School of Physics and Astronomy, Shanghai Jiao Tong University, Shanghai 200240, China*

[2] *ICFO-Institut de Ciencies Fotoniques, The Barcelona Institute of Science and Technology, 08860 Castelldefels (Barcelona), Spain*

[3] *Institute of Spectroscopy, Russian Academy of Sciences, Troitsk, Moscow, 108840, Russia*

[4] *Universitat Politecnica de Catalunya, 08034 Barcelona, Spain*

[5] *Departamento de Física, and Centro de Física Teórica e Computacional, Faculdade de Ciências, Universidade de Lisboa, Campo Grande, Ed. C8, Lisboa 1749-016, Portugal*

[*] *Corresponding author: fangweiye@sjtu.edu.cn*

(Dated: October 17, 2022)

This PDF file includes:

Supplementary Text

Supplementary Figs.1 to 6

Supplementary Text

Configuration of laser beams and tilted moiré lattice. The tilted moiré pattern used in the experiment was created by two interfering lattice-forming TE beams, each one formed by four plane waves:

$$E = \frac{\mathcal{E}}{2} \sum_{m=1}^{4} \left(e^{i\boldsymbol{k}_m \cdot \boldsymbol{r}} + p e^{i\boldsymbol{k}'_m \cdot \boldsymbol{r}} \right), \tag{1}$$

where the wavevectors $\boldsymbol{k}_m$ are given by

$$\boldsymbol{k}_{1,2} = (\mp k_x \cos\theta, \pm k_x \sin\theta, k_z), \quad \boldsymbol{k}_{3,4} = (\mp k_y \sin\theta, \mp k_y \cos\theta, k_z)$$

and $\boldsymbol{k}'_m = R_x(\alpha) R_z(\theta) \boldsymbol{k}_m$, where $R_\nu(\varphi)$, $\nu = x, z$, stand for the standard operators of 3D rotations through an angle φ with respect to ν-axis and $m = 1, ..., 4$. The amplitude of the waves, $\mathcal{E}$, in our experiment had the value of $\mathcal{E} \sim 460$ V/m. Thus, the lattice intensity in main text Eq. (1), determined by the mechanism of the photorefractive response, is approximated by the optical potential Eq. (2) of main text (where we neglected terms of the order of $\alpha^2 y$ and, $\alpha^3 z$ and all higher orders) with the reference lattice

$$V(\boldsymbol{r}) = \mathcal{E}[\cos(k_x x) + \cos(k_y y)] \tag{2}$$

The $z-$independent primitive translation vectors of the so created photonic lattice at $\alpha > 0$ and $m = 2$ and $n = 1$, used in the experiment, are $\boldsymbol{e}_1 = 2\mathbf{i} - \mathbf{j}$ and $\boldsymbol{e}_2 = \mathbf{i} + 2\mathbf{j}$.

Tight-binding approximation. We assume that a complete basis of well localized 2D Wannier functions (or quasi-Wannier functions) $w_\nu(\boldsymbol{r} - \boldsymbol{e}, z)$ exists [1, 2] (as a matter of fact, this requirement can weakened, since we are dealing only with the upper occupied band, and, say, eventual crossings of other empty bands would not affect the observed effects). Substituting the respective expansion

$$\psi = \sum_{\nu, \boldsymbol{e}} c_{\nu \boldsymbol{e}}(z) w_\nu(\boldsymbol{r} - \boldsymbol{e}, z) \tag{3}$$

in Eq. (1) of the main text, and using the definition of the Wannier functions

$$w_\nu(\boldsymbol{r}-\boldsymbol{e},z)=\frac{L^2}{(2\pi)^2}\int_{BZ}e^{-i\boldsymbol{k}\cdot\boldsymbol{e}}\varphi_{\nu\boldsymbol{k}}(\boldsymbol{r},z)d^2k \tag{4}$$

we obtain

$$i\frac{dc_{\nu\boldsymbol{e}}}{dz}+\sum_{\nu_1,\boldsymbol{e}_1}\mathcal{B}_{\nu\nu_1}(\boldsymbol{e}-\boldsymbol{e}_1,z)c_{\nu_1\boldsymbol{e}_1}+\sum_{\boldsymbol{e}_1}\hat{\varepsilon}_\nu(\boldsymbol{e}-\boldsymbol{e}_1,z)c_{\nu\boldsymbol{e}_1}=0 \tag{5}$$

where

$$\hat{\beta}_\nu(\boldsymbol{e},z)=\frac{L^2}{4\pi^2}\int_{BZ}\beta_{\nu\boldsymbol{k}}(t)e^{-i\boldsymbol{k}\cdot\boldsymbol{e}}d^2k \tag{6}$$

is the Fourier coefficient of the expansion of the propagation constant that is periodic in the reciprocal space, and

$$\mathcal{B}_{\nu\nu_1}(\boldsymbol{e}-\boldsymbol{e}_1,z)=\int w_\nu^*(\boldsymbol{r}-\boldsymbol{e},z)(i\partial_z)w_{\nu_1}(\boldsymbol{r}-\boldsymbol{e}_1,z)d^2r. \tag{7}$$

In the linear case, when only the upper band with $\nu=1$ is excited, i.e., when $c_{\nu\boldsymbol{e}}(z=0)=0$ for all $\nu>1$, all these coefficients remain zero if the evolution is adiabatic and no band crossing occurs (these are condition of our experiments). Furthermore, from (7) one obtains that $\mathcal{B}_{\nu\nu}(\boldsymbol{0},z)\equiv 0$, while $\mathcal{B}_{\nu\nu}(\boldsymbol{e},z)$ with nonzero $\boldsymbol{e}$ is negligible due to the tight localization of the Wannier functions, and has additional smallness due to adiabatic variation of the Wannier functions.

On the other hand, flatness of the bands implies smallness of the Fourier coefficients $\hat{\beta}_\nu(\boldsymbol{e},z)$ at all z. This justifies weak diffraction: recall that the diffraction shown in the main text while being visible, corresponds to extremely long (in dimensionless units) propagation distances. Therefore the quantized pumping of the center of mass is determined by the adiabatic evolution of the Bloch states determining the Chern numbers.

Beam dynamics. Using the Wannier-function expansion (3) it is straightforward to compute the representation $\boldsymbol{r}_{\rm c}=\chi(z)\boldsymbol{R}(z)+\boldsymbol{\rho}_{\rm t}(z)+\boldsymbol{\rho}_{\rm ib}(z)$, used in the main text with the

explicit expression for

$$\boldsymbol{\rho}_{\rm t}(z) = \frac{1}{P}\sum_{\boldsymbol{e}} \boldsymbol{e}|c_{1\boldsymbol{e}}|^2 \tag{8}$$

$$\boldsymbol{\rho}_{\rm ib}(z) = \frac{1}{P}\sum_{\substack{\boldsymbol{e}\neq\boldsymbol{e}'\\ \nu\nu'>1}} \boldsymbol{J}^{\nu\nu'}_{\boldsymbol{e}'\boldsymbol{e}} c^*_{\nu'\boldsymbol{e}'} c_{\nu\boldsymbol{e}} \tag{9}$$

where $\boldsymbol{J}^{\nu'\nu}_{\boldsymbol{e}'\boldsymbol{e}} = \int_{\mathbb{R}^2} w^*_{\nu'}(\boldsymbol{r}-\boldsymbol{e}')\boldsymbol{r}w_\nu(\boldsymbol{r}-\boldsymbol{e})d^2r$ and asterisk stands for the complex conjugation. The first term in this expression describes the transition between the bands while the second term describes the diffraction of the beam due to the energy redistribution in the first upper band (for a highly symmetric input beam, like the one used in experiments this term almost vanishes). The evolution of the vector $\boldsymbol{R}(z)$ for the band $\nu = 1$, can be expressed in terms of the Bloch functions through the Berry connection

$$\boldsymbol{A}(\boldsymbol{k}, z) = \int_{\Omega_L} u^*_{1\boldsymbol{k}}(\boldsymbol{r}, z)(i\nabla_{\boldsymbol{k}})u_{1\boldsymbol{k}}(\boldsymbol{r}, z)d^2r =: \langle u_{1\boldsymbol{k}}|i\nabla_{\boldsymbol{k}}u_{1\boldsymbol{k}}\rangle \tag{10}$$

where Ω_L is the primitive cell of the tilted moiré lattice. One computes

$$\boldsymbol{R}(z) = \frac{L^2}{(2\pi)^2}\int_{BZ} \boldsymbol{A}(\boldsymbol{k}, z)d^2k \tag{11}$$

where BZ stands for the reduced Brillouin zone of the tilted moiré lattice. The Bloch functions are normalized to one: $\langle u_{\nu\boldsymbol{k}}|u_{\nu\boldsymbol{k}}\rangle = 1$.

Assuming that $\boldsymbol{R}(0) = 0$, we obtain the shift after one period Z as $\boldsymbol{R}(Z)$. For the Pythagorean lattice used in the experiment ($m = 2$ and $n = 1$) we have $|\boldsymbol{e}_1| = |\boldsymbol{e}_2| = \sqrt{5}a$ and in the system of coordinates defined by the translation vectors $\boldsymbol{e}_{1,2}$ we compute $\boldsymbol{R}(Z) = C_1\boldsymbol{e}_1 + C_2\boldsymbol{e}_2$ with $C_1 = -1$ and $C_2 = 1$ (computed numerically).

Tilted moiré lattices with twisting angle corresponding to $(5, 12, 13)$**.** Moiré lattices used in our experiment were constructed using the first Pythagorean triple $(3, 4, 5)$. This ensures that the first finite gap in the spectrum of this lattice remains open at all distances z. This lattice also has the smallest primitive cell as compared to other square Pythagorean moiré lattices. However, tilted Pythagorean moiré lattices corresponding to other triples may exhibit gap closing at specific propagation distances z. This unwanted effect may preclude observation of topological transport due to coupling between different

bands. In Supplementary Fig. 2 we illustrate this transformation of bandgap structure for a lattice associated with the triple (5,12,13) ($m = 3$ and $n = 2$).

Light propagation in three different types of lattices. Supplementary Fig. 3 shown below compares the evolution of light beam in three different types of lattices, which are: (a) moiré lattice used in our experiment and created by two mutually twisted and tilted sublattices, (b) usual periodic lattice created by two non-twisted and non-tilted sublattices, i.e. lattice obtained at $\theta = \alpha = 0$, (c) the lattice created by two tilted, but non-twisted sublattices at $\theta = 0$ and $\alpha = 0.015$. In all three cases the same input Gaussian beam $\psi(\boldsymbol{r}) = \exp(-r^2/r_0^2)$ of width $r_0 = 0.7$, covering approximately one cell of the lattice, was applied (the beam is illustrated by the red spot superimposed on the lattice profile in the first column). One can readily make two observations. First, light diffraction is significantly suppressed in the moiré lattice (Supplementary Fig. 3a). In this lattice even the pattern after one or two full pumping cycles is narrower than diffraction patterns in two other lattices at a quarter and a half cycle (their quantitative comparison is shown in Fig.2h of the main text). Second, no directed displacement of the light pattern is seen in the lattice produced by tilted, but non-twisted sublattices (Supplementary Fig. 3c). Propagation up to larger distances z in this type of lattice confirms this observation.

Characterization of the beam displacement. To characterize the displacement of the light beam we used the well-defined coordinate of the center of mass (COM), which accounts for the total integral intensity of the light beam. The COM trajectory, shown by the green curve in Supplementary Fig. 4b, predicts somewhat smaller displacement after one pumping cycle in comparison with ideal pumping (which is obtained also in Fig.2d and Fig.3b of the main text). Such deviation from the ideal pumping is mainly due to the diffraction of the pattern. Indeed, if the small-amplitude diffracted field is discarded upon calculation of the COM, then nearly perfect shift is obtained. This is illustrated by the red curve in Supplementary Fig. 4b, where upon calculation of COM we discarded the contribution of the field below 9% of the peak intensity. Furthermore, if the displacement of the pattern is characterized by the position of the global intensity maximum (blue curve of Supplementary Fig. 4b), we again observe oscillations of displacement around the idealized curve, but at the same time a perfect match with the idealized pumping is obtained after a full pumping cycle. In this case, the position of the main lobe of the diffraction pattern after one pumping cycle matches exactly the position predicted by the idealized theory, as

shown in Supplementary Fig. 4a.

2D Thouless pumping for negative twisting angle. In the main text, we present the pumping of the light when the twisting angle θ between two sublattices is equal to $\theta = \arctan(2mn/(m^2 - n^2)) = \arctan(4/3)$ with integer $m = 2$ and $n = 1$. In this case the two Chern numbers determining pumping direction of light are given by $(C_1, C_2) = (-1, 1)$ and in accordance with the formula $\boldsymbol{R}(Z) = C_1\boldsymbol{e}_1 + C_2\boldsymbol{e}_2$, the light exhibits pump into the second quadrant (Supplementary Fig. 5a). If, on the other hand, the twisting angle θ changes its sign, $\theta = -\arctan(4/3)$, that corresponds to $(m, n) = (2, -1)$, the above Chern numbers become $(C_1, C_2) = (1, 1)$, and the light is found to pump into the first quadrant (Supplementary Fig. 5b). Direct comparison between θ and $-\theta$ cases shows that the magnitude of the COM displacement is exactly the same in both cases, but the direction of displacement changes from $+18^o$ to -18° with respect to the pumping direction of the lattice. Intensity distributions after one and two pumping cycles for these two twisting angles are depicted in Supplementary Fig. 5c and 5d.

Approaching the adiabatic regime of the Thouless pumping. A perfect pumping is only achieved when the pumping cycle (z-period of the lattice $Z = 2a/\alpha$) tends to infinity. In reality, the sample length is finite (2 cm long in our case) that imposes a practical limitation with respect to how close one can approach the adiabaticity. In our experiment we used an angle $\alpha = 0.015$, yielding lattice period of 1.2 cm, which is of course not very close to the mathematical adiabatic limit, but still, we observed an excellent agreement between the direction of the beam pumping and prediction based on the topology of the system. This confirms that we are working in the quasi-adiabatic regime. To illustrate how pumping dynamics depends on the angle α (z-period of the lattice) and to give an idea on transition to adiabatic regime, in supplementary Figure 6 we show the results of simulations of the displacement of the beam center of mass upon pumping in moiré lattice for several progressively decreasing angles α (results for two such angles are also included into main text in Fig. 2) at fixed dc field E_0. These results clearly show that pumping regime becomes nearly adiabatic (i.e. COM displacement for light beam practically exactly coincides with prediction based on the Chern numbers) in samples with lengths $\sim$ 5-10 cm that in principle can be manufactured. On the other hand, when cyclic modulation becomes too fast, it would result in significant inter-band transitions. In this regime the direction/magnitude of displacement of the beam depends on the details of modulation (rather than determined by

topological indices of the bands), as is well explained in [3].

[1] C. Brouder, G. Panati, M. Calandra, C. Mourougane, N. Marzar, Exponential Localization of Wannier Functions in Insulators, *Phys. Rev. Lett.* **98**, 046402 (2007).

[2] N. Marzari, A. A. Mostof, J. R. Yates, I. Souza, D. Vanderbilt, Maximally localized Wannier functions: Theory and applications, *Rev. Mod. Phys.* **84**, 1419-1475 (2012).

[3] L. Privitera, A. Russomanno, R. Citro, G. E. Santoro, Nonadiabatic Breaking of Topological Pumping. *Phys. Rev. Lett.* **120**, 106601 (2018).

Figures

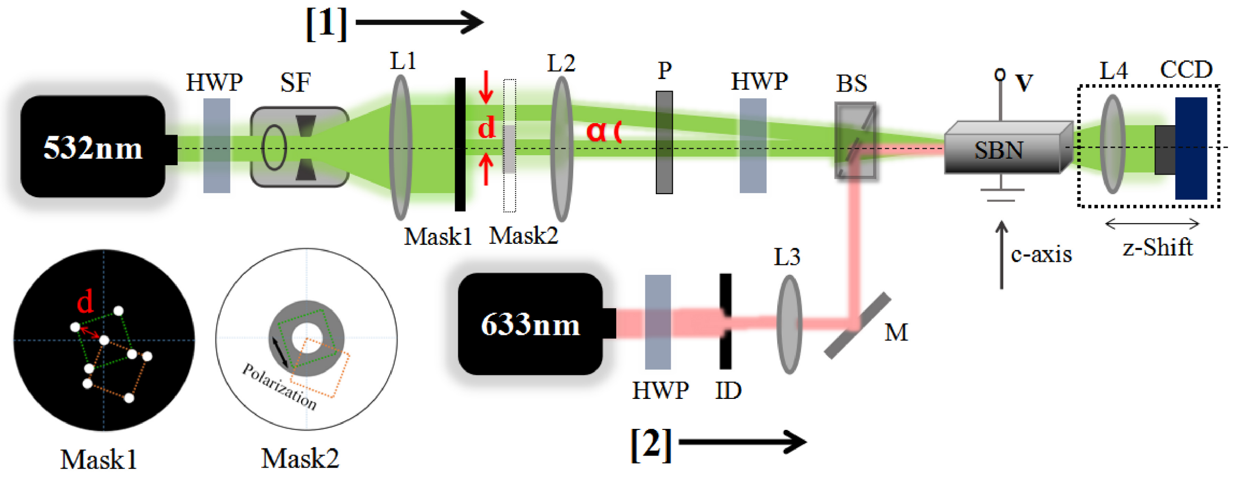

Supplementary Fig. 1: **Experimental setup.** HWP, half-wave plate; SF, spatial filter; L, lens; P, polarizer; ID, iris diaphragm; BS, beam splitter; SBN, strontium barium niobate crystal; CCD, charged-coupled device. Mask 1 is an amplitude mask used to produce two group of sub-lattices with rotation angle θ, and mask 2 is made of a polarizer film.

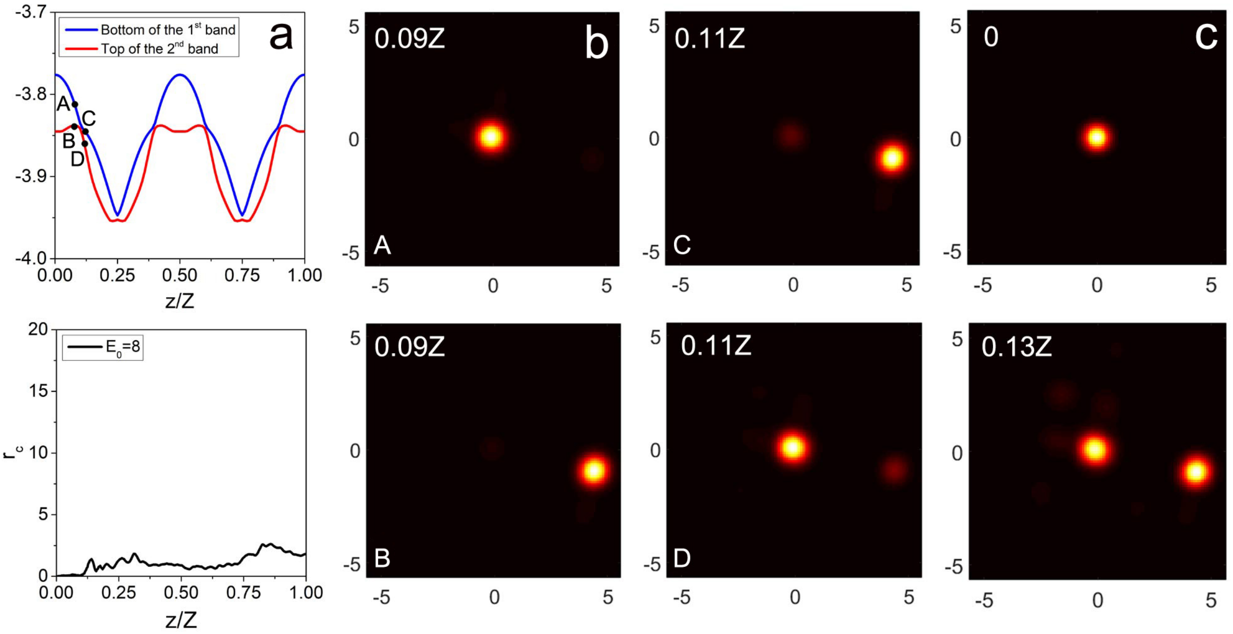

Supplementary Fig. 2: **Tilted moiré lattice with band crossing** for the Pythagorean angle $\theta = \arctan(12/5)$ and tilt angle $\alpha = 0.002$. (**a**) Evolution of the bottom of the first band (blue line) and the top of the second band (red line) with distance z. Bottom: Pumping in the lattice does not show overall displacement of the COM of the light beam. (**b**) Representative Bloch mode profiles at a distance z taken just before (A, B) and after (C, D) the point, where gap closes. Note the mode swap before and after gap closure. (**c**) Evolution of light intensity at $z = 0$ (before gap closure) and at $z = 0.13Z$ (after gap closure), showing state transformation from single-humped into double-humped as a consequence of the band inversion.

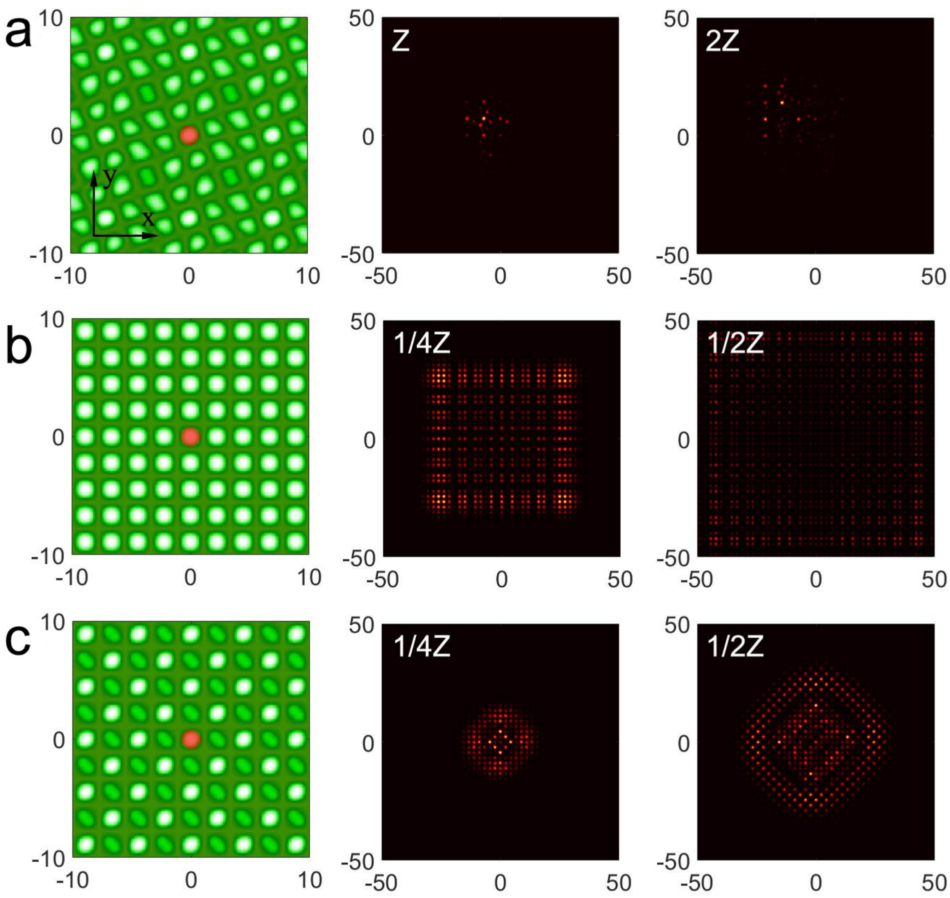

Supplementary Fig. 3: **Propagation of light in three types of superlattices.** Propagation of light inside a tilted moiré lattice (**a**, first row), a single reference lattice (**b**, second row) of the depth $p = 1.3$, and a tilted lattice without twist for sublattice depths of 1 and 0.3, at $\alpha = 0.015$ (**c**, third row). The first column shows lattice profiles at $z = 0$, with the red dots indicating the position of the input light. Notice different propagation distances for distributions shown: $2Z$ in the tilted moiré lattice (upper row) and $1/2Z$ in the middle and bottom rows.

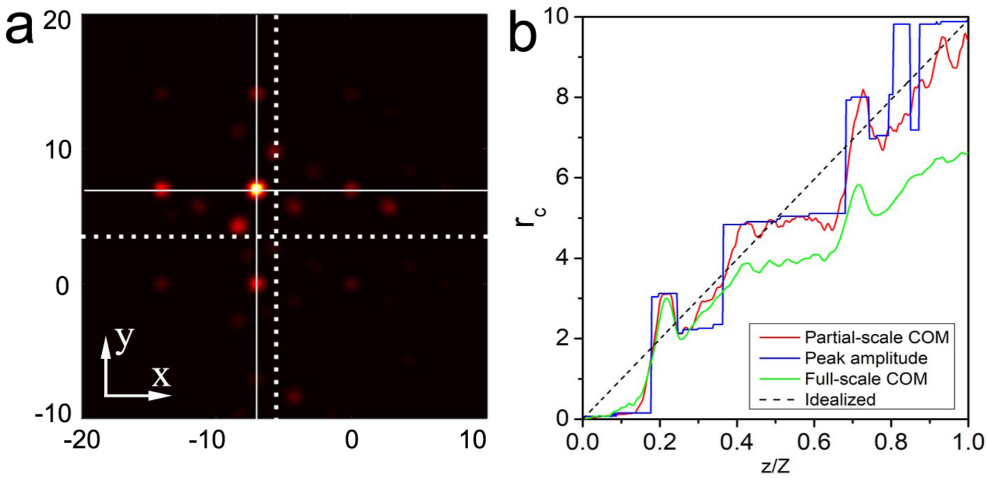

Supplementary Fig. 4: **Characterization of the displacement of the beam for Thouless pumping.** **a**, The field distribution after one pumping cycle in the tilted moiré lattice. The crossing point of the solid lines indicates the prediction of theory, while the crossing point of the dashed lines indicates the location of the full-scale COM. **b**, The displacement of the wavepacket *versus* z: the green curve (Full-scale COM) is the COM that is computed taking into account the entire intensity distribution; the red curve (Partial-scale COM) is the COM that is computed by discarding small-intensity background (regions with intensity below 9% of the peak intensity are not taken into account); the blue curve is the position of the global intensity maximum.

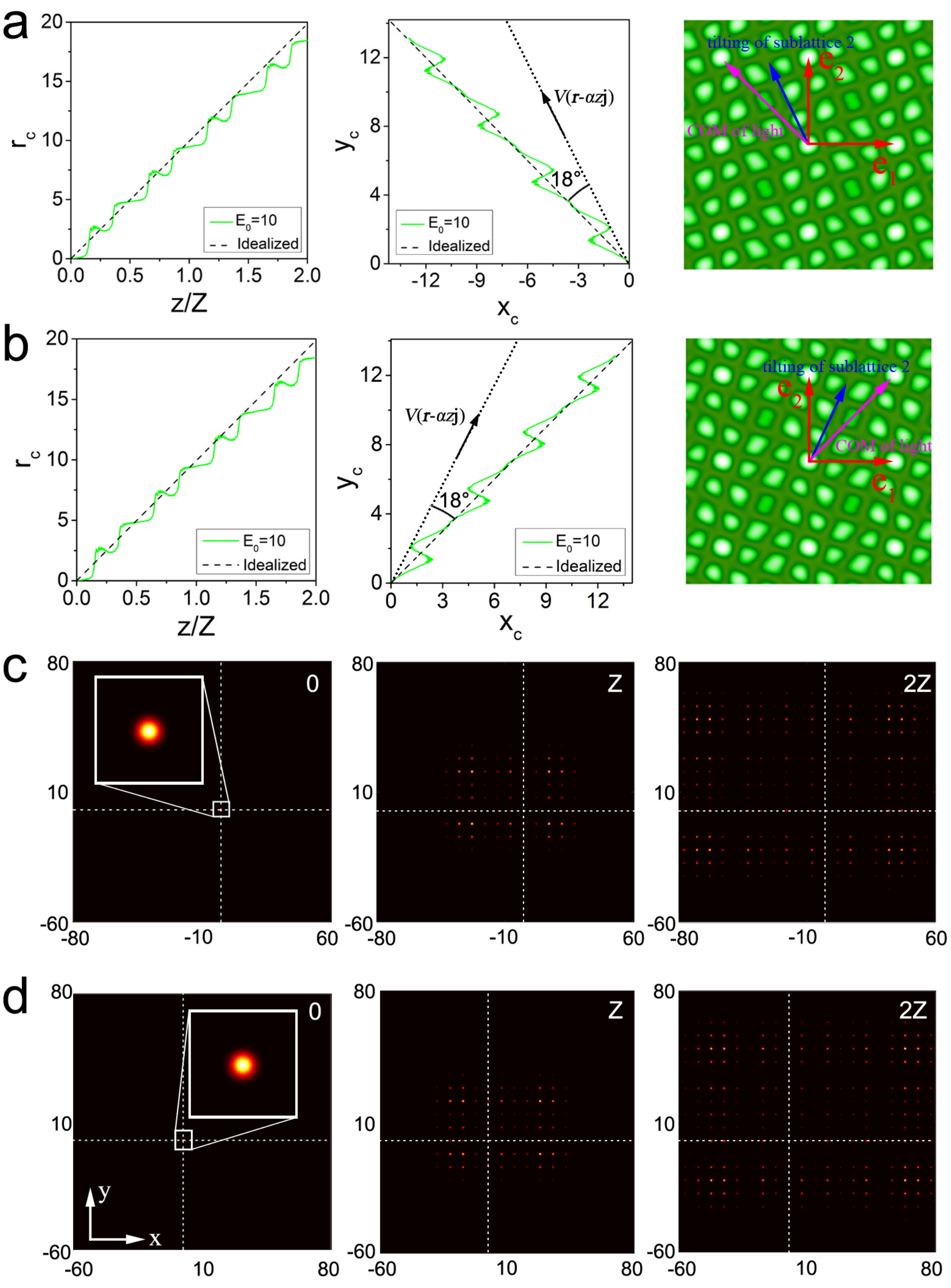

Supplementary Fig. 5: **Direct comparison of Thouless pumping of light in two sliding moiré lattices with opposite twisting angles**, $\theta = \arctan(4/3)$ (**a, c**) and $\theta = -\arctan(4/3)$ (**b, d**). (**a, b**): The first and second panels show respectively the displacements of the light beam COM, $|\boldsymbol{r}_c|$, and the trajectories of the COM $\boldsymbol{r}_{\rm c} = (x_{\rm c}, y_{\rm c})$ with distance z in the transverse plane. The third panel is the schematic plot showing moiré lattice profile at $z = 0$ and the direction of the pumping of the lattices (blue arrow) as well as the pumping of the light (magenta arrow). (**c, d**): Snapshots of beam propagation at distances $z = 0, Z, 2Z$ (zoom of the initial intensity distribution is shown within white square). In all cases shown $\alpha = 0.002$ and $E_0 = 10$.

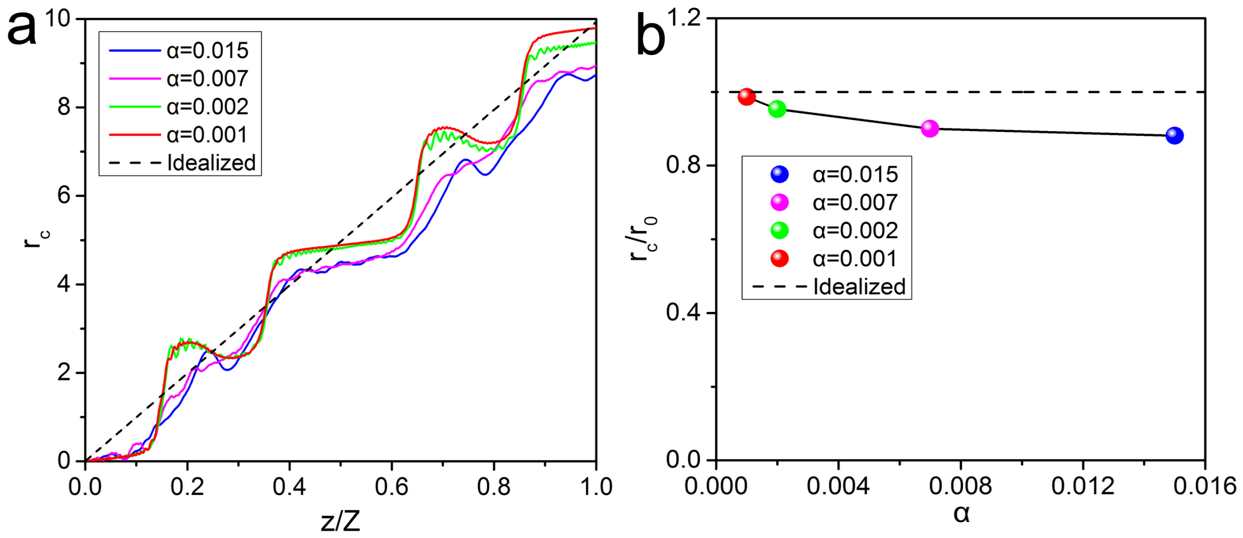

Supplementary Fig. 6: **Approaching adiabatic regime with decreasing tilt angle α.** (**a**) The displacement of the COM of the wavepacket versus z for $\alpha = 0.015$ (blue), 0.007 (magenta), 0.002 (green) and 0.001 (red). In all cases $E_0 = 8$. (**b**) The displacement of the COM of the wavepacket after one Z-period, for four values of α as in (**a**). Dashed line in (**a**) and (**b**) represents the idealized displacement.
